# Positive end-expiratory pressure at minimal respiratory elastance represents the best compromise between mechanical stress and lung aeration in oleic acid induced lung injury

**DOI:** 10.1186/cc6093

**Published:** 2007-08-09

**Authors:** Alysson Roncally S Carvalho, Frederico C Jandre, Alexandre V Pino, Fernando A Bozza, Jorge Salluh, Rosana Rodrigues, Fabio O Ascoli, Antonio Giannella-Neto

**Affiliations:** 1 Biomedical Engineering Program, COPPE, Federal University of Rio de Janeiro, Av. Horácio Macedo, CT Bloco H-327, 2030, 21941-914, Rio de Janeiro, Brazil; 2 Fundação Oswaldo Cruz, Instituto de Pesquisa Clinica Evandro Chagas e Laboratório de Imunofarmacologia, IOC, Av Brasil, 4365, Manguinhos, 21045-900 Rio de Janeiro, Brazil; 3 National Institute of Cancer-1, ICU, Praça Cruz Vermelha, 20230-130 Rio de Janeiro, Brazil; 4 Radiodiagnostic Service, Clementino Fraga Filho Hospital, Federal University of Rio de Janeiro, R Professor Rodolpho Paulo Rocco, 255, 21-941-913 Rio de Janeiro, Brazil

## Abstract

**Introduction:**

Protective ventilatory strategies have been applied to prevent ventilator-induced lung injury in patients with acute lung injury (ALI). However, adjustment of positive end-expiratory pressure (PEEP) to avoid alveolar de-recruitment and hyperinflation remains difficult. An alternative is to set the PEEP based on minimizing respiratory system elastance (Ers) by titrating PEEP. In the present study we evaluate the distribution of lung aeration (assessed using computed tomography scanning) and the behaviour of Ers in a porcine model of ALI, during a descending PEEP titration manoeuvre with a protective low tidal volume.

**Methods:**

PEEP titration (from 26 to 0 cmH_2_O, with a tidal volume of 6 to 7 ml/kg) was performed, following a recruitment manoeuvre. At each PEEP, helical computed tomography scans of juxta-diaphragmatic parts of the lower lobes were obtained during end-expiratory and end-inspiratory pauses in six piglets with ALI induced by oleic acid. The distribution of the lung compartments (hyperinflated, normally aerated, poorly aerated and non-aerated areas) was determined and the Ers was estimated on a breath-by-breath basis from the equation of motion of the respiratory system using the least-squares method.

**Results:**

Progressive reduction in PEEP from 26 cmH_2_O to the PEEP at which the minimum Ers was observed improved poorly aerated areas, with a proportional reduction in hyperinflated areas. Also, the distribution of normally aerated areas remained steady over this interval, with no changes in non-aerated areas. The PEEP at which minimal Ers occurred corresponded to the greatest amount of normally aerated areas, with lesser hyperinflated, and poorly and non-aerated areas. Levels of PEEP below that at which minimal Ers was observed increased poorly and non-aerated areas, with concomitant reductions in normally inflated and hyperinflated areas.

**Conclusion:**

The PEEP at which minimal Ers occurred, obtained by descending PEEP titration with a protective low tidal volume, corresponded to the greatest amount of normally aerated areas, with lesser collapsed and hyperinflated areas. The institution of high levels of PEEP reduced poorly aerated areas but enlarged hyperinflated ones. Reduction in PEEP consistently enhanced poorly or non-aerated areas as well as tidal re-aeration. Hence, monitoring respiratory mechanics during a PEEP titration procedure may be a useful adjunct to optimize lung aeration.

## Introduction

Mechanical ventilation has become the most important life support modality in patients suffering from acute lung injury (ALI) [1]. However, use of high tidal volumes (V_T_s) and inappropriate levels of positive end-expiratory pressure (PEEP) may worsen any pre-existing lung inflammatory process [2,3].

Currently, a major difficulty when instituting a lung-protective ventilatory strategy in ALI lies in the objective determination of a PEEP level that prevents alveolar de-recruitment without inducing lung over-inflation and pulmonary distortion [4-6]. In clinical practice PEEP is usually adjusted according to oxygenation response and the required fraction of oxygen [7], but both PEEP-induced over-distension and tidal recruitment are rather difficult to detect [8]. An alternative is to determine an 'optimal' level of PEEP based on minimizing the mechanical stress that results from tidal alveolar recruitment and over-distension [9]. For this purpose, the deflation limb of the pressure-volume curve has been used to identify the level of PEEP that effectively prevents alveolar de-recruitment [7,10]. However, pressure-volume curves are not easily obtained at the bedside and often require special manoeuvres, such as disconnection from the ventilator or modifications to the tidal ventilatory pattern.

Morphological analysis of lung computed tomography (CT) images has been used to assess lung aeration, and this approach may provide an objective tool with which to establish optimal mechanical ventilation settings [11-14]. However, the CT scan is not portable and often requires transport of the patient to the radiology department.

A clinically feasible alternative is to set the PEEP level based on minimizing the elastance of the respiratory system (Ers), during a descending PEEP titration [15,16]. In healthy piglets managed using a protective low V_T _ventilatory strategy, we recently showed that the PEEP at which the minimum Ers was observed (PEEP_Ers_) appeared to represent a good compromise between maximum lung aeration and least areas of hyperinflation and de-recruitment [17]. Similarly, it has been shown that continuous monitoring of the dynamic respiratory system compliance permitted the detection of alveolar de-recruitment in a protocol involving descending PEEP titration in a surfactant-depleted swine model [18].

The aim of this work was to evaluate the distribution of lung aeration, as assessed based on morphological analysis of CT images, and the behaviour of the Ers in a porcine model of ALI, during a descending PEEP titration manoeuvre with a low V_T_. The correspondence and contrast between Ers and distribution of lung aeration, particularly the distribution of lung aeration at PEEP_Ers_, were examined. In addition, the feasibility of using continuous monitoring of the Ers to establish the optimal PEEP level is discussed.

## Materials and methods

The protocol was submitted and approved by the local Ethics Committee for Assessment of Animal Use in Research (CEUA/FIOCRUZ).

### Animal preparation

The animal preparation and protocol, apart from ALI induction, were similar to those presented in detail in the report by Carvalho and coworkers [17]. In brief, six piglets (17 to 20 kg), laying in the supine position, were pre-medicated with midazolam (Dormire; Cristália, São Paulo, Brazil) and connected to an Amadeus ventilator (Hamilton Medical; Rhäzüns, Switzerland). The animals underwent volume-controlled ventilation with square flow waveform, with a PEEP of 5 cmH_2_O, fractional inspired oxygen of 1.0, V_T _of 8 ml/kg, inspiratory/expiratory ratio of 1:2, and respiratory rate between 25 and 30 breaths/min, in order to maintain normocapnia (arterial carbon dioxide tension range 35 to 45 mmHg). A flexible catheter was inserted through which blood samples were drawn for blood gas analysis (I-STAT with EG7+ cartridges; i-STAT Corp, East Windsor, USA) in order to certify that ALI criteria were satisfied. The animals were sedated with a continuous infusion of ketamine (Ketamina; Cristália) delivered at a rate of 10 mg/kg per hour and paralyzed with pancuronium (Pavulon; Organon Teknika, São Paulo, Brazil) at 2 mg/kg per hour. The airway opening pressure was measured using a pressure transducer (163PC01D48; Honeywell Ltd, Freeport, USA) connected to the endotracheal tube, and flow was measured using a variable-orifice pneumotachometer (Hamilton Medical) connected to a pressure transducer (176PC07HD2; Honeywell Ltd). Airway opening pressure and flow were digitized at a sampling rate of 200 Hz per channel. The volume was calculated by numerical integration of flow.

### Experimental protocol

**Figure 1:**
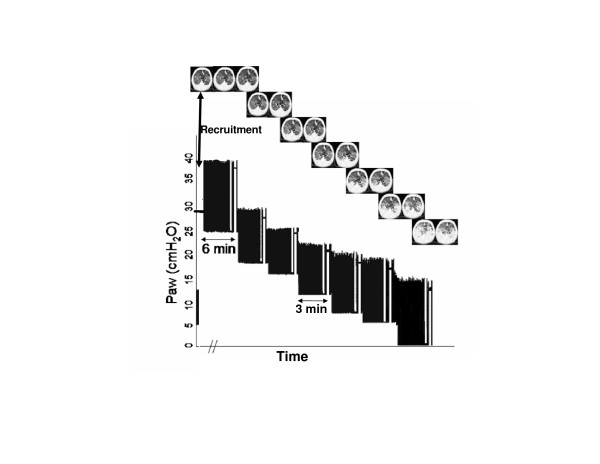
Time plot of Paw during the PEEP titration procedure. The baseline ventilation, with a PEEP of 5 cmH_2_O, and the recruitment maneuver followed by the descending PEEP titration are shown. At the end of each PEEP step, a CT scan was performed at end-expiratory (left) and end-inspiratory (right) pauses. (CT scan images from a representative animal are shown.) CT, computed tomography; Paw, airway opening pressure; PEEP, positive end-expiratory pressure.

After 20 to 120 min of artificial ventilation, lung injury was induced by means of central venous infusion of oleic acid (0.05 ml/kg) until the arterial oxygen tension (PaO_2_) fell to below 200 mmHg for at least 30 min. After lung injury was established, the V_T _was set to 6 ml/kg and a recruitment manoeuvre was performed, with a sustained inflation of 30 cmH_2_O over 30 s. The PEEP was titrated by descending from 26 cmH_2_O to 20, 16, 12, 8, 6 and then 0 cmH_2_O (zero PEEP [ZEEP]). The duration of each step was 3 min, except for the 26 cmH_2_O step and ZEEP (6 min each; Figure 1). All mechanical ventilation parameters were kept constant during the entire titration procedure. At the end of the protocol, the animals were killed using an intravenous injection of potassium chloride while they were deeply sedated.

### Computed tomography scan procedure and image analysis

Helical CT scans (Asteion; Toshiba, Tokyo, Japan) were obtained at a fixed anatomic level in the lower lobes of the lungs, corresponding to the greatest transverse lung area. Each scan comprised five to seven thin section slices (1 mm). Scanning time, tube current and voltage were 1 s, 120 mA and 140 kV, respectively. The actual image matrix was 512 × 512 and the voxel dimensions ranged from 0.22 to 0.29 mm. The scans were obtained at the end of each PEEP step, during end-expiratory and end-inspiratory pauses of 15 to 20 s (Figure 1). All images were acquired with the animal laying supine position during the entire protocol.

The images were imported and analyzed using a purpose-built routine (COPPE-CT) written in MatLab (Mathworks, Natick, MA, USA). The lung contours were manually traced to define the region of interest. The presence of hyperinflated (-1,000 to -900 Hounsfield units, coloured in red), normally aerated (-900 to -500 Hounsfield units, blue), poorly aerated (-500 to -100 Hounsfield units, light grey) and non-aerated areas (-100 to +100 Hounsfield units, dark grey) was determined, in accordance with a previously proposed classification [14,19]. The absolute weight of tissue (in grams) in each slice as well as in each compartment within the slice was also calculated using standard equations [14]. Attenuation values outside the range of -1,000 to +100, which contributed under 1% of all counts, were excluded. In order to compare the images obtained at end-expiration and end-inspiration, the slices with the greatest anatomical coincidence between end-expiration and end-inspiration images were chosen, by selecting one of the last five to seven slices at end-expiration and one of the first slices at the end-inspiration.

In order to evaluate any possible cephalo-caudal gradient, in two of the animals three CT scan slices were obtained at the apical level (near hilus), middle (near the carina) and basal (up to diaphragm) at a PEEP of 26 cmH_2_O during end-expiratory and end-inspiratory pauses.

### Data analysis

Signals of airway opening pressure, flow and volume were used to obtain the parameters required by the equation of motion of the respiratory system using least-squares linear regression, considering a linear single-compartment model:

Paw = Ers × V(t) + Rrs × dV(t)/dt + EEP

Where Rrs is the resistance of the respiratory system, V(t) is the volume, dV/dt is the flow and EEP is the end-expiratory pressure. Curve fitting to the linear single-compartment model (Eqn 1) was performed using data acquired during the entire PEEP titration procedure. For data analysis, mean values of Ers, Rrs and EEP were calculated on a breath-by-breath basis from the last minute of each PEEP step, and immediately before the CT scanning was performed. The quality of fitting was assessed using the coefficient of determination of the regression (R^2^).

### Statistical analysis

Data are presented as median and range values, attributed to the respective PEEP values. The peak and plateau pressures, as well as the estimated and applied PEEP values, were measured at each PEEP level. A Wilcoxon signed rank test for paired samples was applied to compare changes in Ers for each PEEP step, as well as changes in lung aeration between end-expiration and end-inspiration at each PEEP value. In all tests, a *P *< 0.05 was considered significant.

## Results

**Figure 2:**
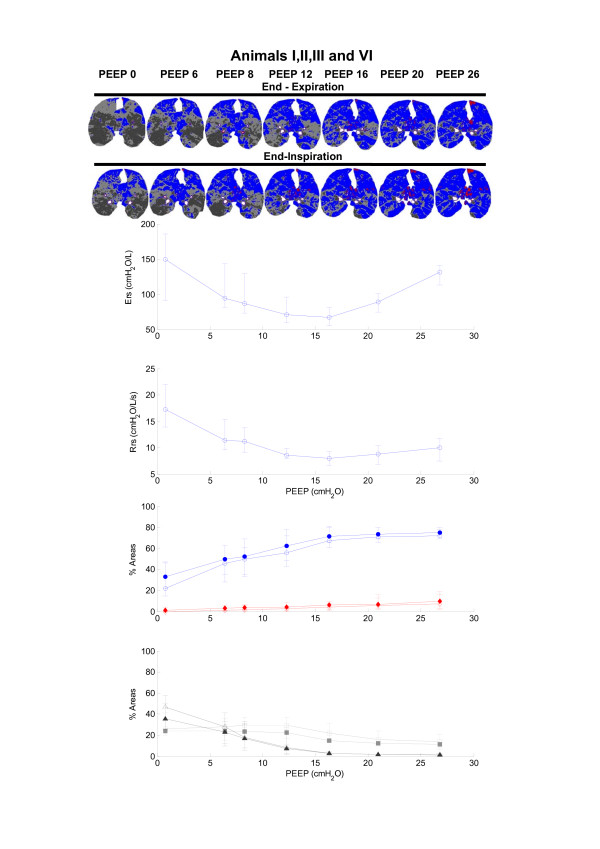
Ers, Rrs and morphological analysis of the CT scans during PEEP titration for animals I, II, III and VI. The median and range of Ers and Rrs, and the distribution of lung aeration are plotted as a function of PEEP. Red diamonds indicate hyperinflated areas, blue circles indicate normally aerated areas, light grey squares indicate poorly aerated areas, and black triangles indicate non-aerated areas. The filled and open symbols indicate lung aeration changes at end-inspiration and end-expiration, respectively. Regions of interest on the CT scan images obtained during the PEEP titration in a representative case (animal I) are also presented in the upper panel. Aeration titration in a representative case (animal I) are also presented in the upper panel. Aeration status is colour coded in the images. Red indicates hyperinflated areas, and blue, light grey and black indicate normally aerated, poorly aerated and non-aerated areas, respectively. CT, computed tomography; Ers, respiratory system elastance; PEEP, positive end-expiratory pressure; Rrs, respiratory system resistance.

**Figure 3:**
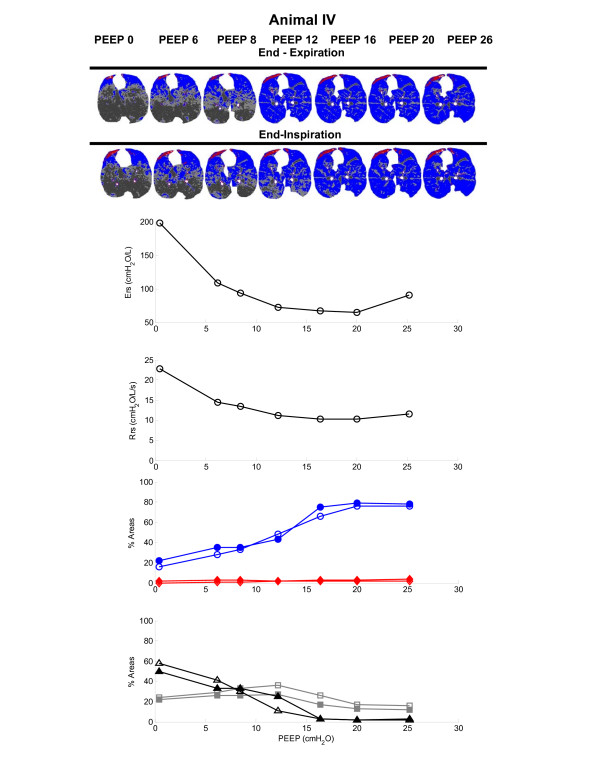
Ers, Rrs and morphological analysis of the CT scans during PEEP titration for animal IV. The regions of interest of the CT scan images obtained during the PEEP titration are also shown in the upper panel. For details, see legend to Figure 2. CT, computed tomography; Ers, respiratory system elastance; PEEP, positive end-expiratory pressure; Rrs, respiratory system resistance.

**Figure 4:**
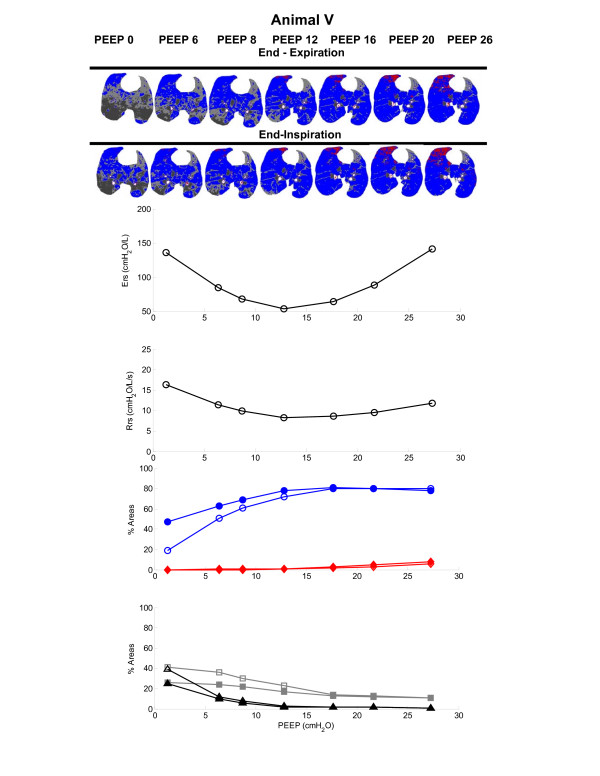
Ers, Rrs and morphological analysis of the CT scans during PEEP titration for animal V. The regions of interest of the CT scan images obtained during the PEEP titration are also shown in the upper panel. For details, see legend to Figure 2. CT, computed tomography; Ers, respiratory system elastance; PEEP, positive end-expiratory pressure; Rrs, respiratory system resistance.

The respiratory mechanics parameters, namely the estimated Ers and Rrs, and the PEEP, are presented in Table 1. The Ers reached a minimum with PEEP set to 16 cmH_2_O for all (Figure 2) but two animals (for which the levels of PEEP that yielded the lowest Ers were 12 cmH_2_O and 20 cmH_2_O; see Figures 3 and 4).

**Table 1:** Respiratory mechanics and regression parameters

Parameter	Descending PEEP titration steps						
PEEP_appl _(cmH_2_O)	27.1 (25.3–27.7)	21.0 (19.8–22.1)	16.3 (15.6–17.2)	12.3 (12–13.1)	8.4 (7.7–9.2)	6.2 (5.9–6.9)	0.8 (0.5–1.7)

Ppeak (cmH_2_O)	47.85 (40–52)	36.4 (31.3–40.5)	29.35 (25.7–30.6)	25.1 (22.6–28.2)	24.05 (21.3–26.4)	23.7 (20.7–25.7)	24 (21.6–28.8)
Pplateau (cmH_2_O)	39.5 (33.8–45.6)	31.2 (28.9–37.5)	25.6 (24.4–28.1)	21.3 (19.2–25.1)	19.1 (17–22.4)	18.1 (16.3–22.2)	17.9 (14.2–21.4)
Ers (cmH_2_O.l^-1^)	131.4 (90.1–141.4)	84.0 (65.1–101)	65.5 (54.9–81.5)	70.4 (53–95.9)	86.4 (67.5–129.2)	94.3 (81.2–143.6)	148.8 (91.2–198)
Rrs (cmH_2_O.l^-1^.s)	11.5 (7.4–11.8)	9.7 (6.8–10.4)	8.7 (6.6–10.3)	8.7 (7.8–11.2)	11.2 (9.1–13.8)	11.7 (9.6–15.3)	17.2 (13.9–22.8)
PEEP_est _(cmH_2_O)	26.7 (25.2–27.7)	20.9 (119.6–20.8)	16.4 (15.4–17.2)	12.3 (12.1–12.6)	8.4 (7.8–8.7)	6.2 (5.88–6.6)	0.45 (0.07–2.2)
R^2^	0.975 (0.97–0.985)	0.975 (0.97–0.983)	0.979 (0.97–0.985)	0.98 (0.97–0.988)	0.98 (0.97–0.99)	0.982 (0.97–0.99)	0.99 (0.88–0.99)

Data are presented as median (range). Ers, elastance of the respiratory system; PEEP, positive end-expiratory pressure; PEEP_appl_, applied PEEP; PEEP_est_, estimated PEEP; P_peak_, peak ventilator pressure; P_plateau_, plateau ventilator pressure; Rrs, resistance of the respiratory system; R^2^, coefficient of determination of the regression analysis.

Table 2 presents the absolute weight of tissue (in grams) at end-expiration and end-inspiration, in each slice and in each compartment within the slice, during the PEEP titration. Note that an overall increase in the slice mass was observed as PEEP decreased. Additionally, a reduction in the slice mass was consistently observed from expiration to inspiration. The slice mass increase was concentrated in the poorly and non-aerated compartments.

**Table 2:** CT-scan slice mass during PEEP titration procedure

Parameter	Descending PEEP titration steps						
PEEP_appl _(cmH_2_O)	27.1 (25.3–27.7)	21.0 (19.8–22.1)	16.3 (15.6–17.2)	12.3 (12–13.1)	8.4 (7.7–9.2)	6.2 (5.9–6.9)	0.8 (0.5–1.7)

Slice mass (g)							
Exp	4.8 (3.0–5.1)	4.9 (3.2–5.1)	5.3 (3.5–5.8)	6.0 (4.0–6.3)	6.7 (4.5–7.9)	7.4 (4.9–9.2)	8.6 (6.4–10.2)
Ins	4.4 (2.9–4.8)	4.6 (3.1–5.2)	4.9 (3.2–5.4)	5.5 (3.4–7.0)	6.2 (3.9–7.0)	6.6 (4.3–8.3)	7.5 (5.1–9.1)
Hyperinflated (g)							
Exp	0.06 (0.02–0.12)	0.06 (0.01–0.1)	0.04 (0.01–0.08)	0.03 (0.00–0.05)	0.02 (0.00–0.06)	0.01 (0.00–0.03)	0.00 (0.00–0.00)
Ins	0.087 (0.03–0.15)	0.07 (0.03–0.12)	0.05 (0.02–0.10)	0.03 (0.01–0.07)	0.03 (0.01–0.06)	0.03 (0.01–0.06)	0.01 (0.00–0.04)
Normally (g)							
Exp	2.7 (1.9–3.2)	2.8 (2.1–3.4)	2.61 (2.2–3.1)	2.16 (1.8–2.5)	1.79 (1.4–2.1)	1.59 (1.2–1.9)	0.83 (0.5–1.4)
Ins	2.67 (1.9–3.14)	2.69 (2.0–3.3)	2.73 (2.1–3.2)	2.30 (1.9–2.5)	1.89 (1.6–2.3)	1.70 (1.4–2.0)	1.15 (0.9–1.3)
Poorly (g)							
Exp	1.4 (0.8–1.7)	1.3 (0.9–1.9)	2.0 (1.0–2.4)	2.7 (1.5–3.1)	2.5 (1.5–3.0)	2.3 (1.8–2.8)	2.3 (2.0–2.9)
Ins	1.0 (0.8–1.5)	1.3 (0.8–1.6)	1.5 (0.9–1.8)	2.1 (1.1–2.4)	2.0 (1.3–2.5)	1.9 (1.2–2.4)	2.0 (1.6–3.0)
Non-aerated (g)							
Exp	0.3 (0.2–0.4)	0.3 (0.2–0.7)	0.4 (0.2–0.9)	0.9 (0.3–1.5)	2.3 (0.8–3.5)	3.5 (1.1–5.3)	5.6 (3.2–7.3)
Ins	0.3 (0.1–0.6)	0.3 (0.2–0.8)	0.5 (0.2–0.8)	0.8 (0.3–2.8)	2.3 (0.6–2.8)	3.0 (1.0–4.5)	3.8 (2.2–6.2)

Shown are the slice mass (absolute slice tissue mass, in grams), and the mass in hyperinflated compartments (Hyperinflated), in normally aerated compartments (Normally), in poorly aerated compartments (Poorly) and in the non-aerated compartments (Non-aerated). Data are presented as median (range). CT, computed tomography; Exp, end-expiratory slice; Ins, end-inspiratory slice; PEEP, positive end-expiratory pressure; PEEP_appl_, applied positive end-expiratory pressure.

### CT scan morphological analyses and respiratory mechanics during PEEP titration

The reduction in PEEP from 26 cmH_2_O to PEEP_Ers _significantly increased poorly aerated areas (ranges increase from 8–21% to 14–31% at end-expiration, and from 7–16% to 13–23% at end-inspiration), with no significant change in non-aerated areas, which remained below 5%. Normally aerated areas remained in a plateau ranging from 61% to 80% at end-expiration and from 66% to 81% at end-inspiration, and hyperinflated areas monotonically decreased (ranges decrease from 2–16% to 1–8% at end-expiration, and from 3–19% to 2–10% at end-inspiration). The distribution of aeration at each PEEP step is depicted in Figures 2 to 4. Note that PEEP_Ers _resulted in the best compromise between normally, hyperinflated and non-aerated areas in all studied animals. A predominance of hyperinflated areas in nondependent lung regions was observed, whereas poorly aerated areas appeared to be more diffusely distributed. Non-aerated areas, which were always less than 5%, occurred in dependent regions (Figures 2 to 4, upper panels).

The progressive reduction in PEEP from PEEP_Ers _to ZEEP resulted in a significant increase in non-aerated areas (ranges increased from 2–4% to 26–58% at end-expiratory pause, and from 2–5% to 25–50% at end-inspiratory pause), with concomitant reductions in normal inflation (from 61–80% to 15–46% at end-expiratory pause, and from 66–81% to 22–47% at end-inspiratory pause) and hyperinflation (from 1–8% to 0–1% at end-expiratory pause, and from 2–10% to 0–4% at end-inspiratory pause).

**Figure 5:**
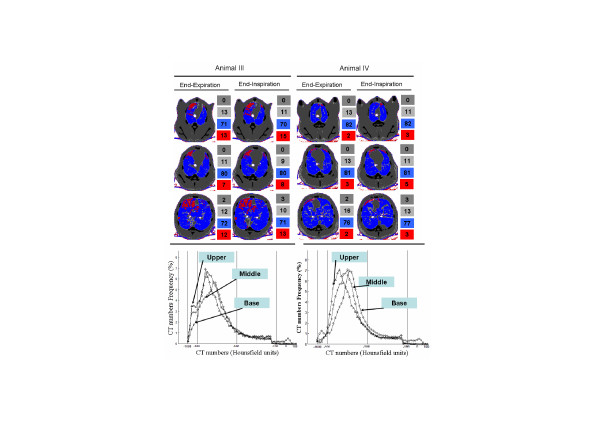
Comparative changes in lung aeration at different anatomic levels. Images from the apex to diaphragm level during an end-expiratory pause and an end-inspiratory pause for two studied animals (left and right columns). The computed tomography (CT) scans were acquired near the lung hilus (upper), near the carina (middle) and at juxta-diaphragmatic (lower) levels; the respective histograms of density are also shown (bottom).

Figure 5 depicts the images and the corresponding density histogram distributions for two animals during end-expiratory and end-inspiratory pauses at a PEEP of 26 cmH_2_O. Note that no significant cephalo-caudal gradient was observed between the apex and basal levels, but in one animal the middle level exhibited less areas of hyperinflation. From the apex to the base, the peak of the histogram shifted toward the normally aerated range (Figure 5, bottom).

## Discussion

### CT scan and elastic properties analysis

The main objective of this work was to assess the correspondence between the findings of CT scan morphological analysis and the dynamics of the mechanical characteristics of the respiratory system, in order to evaluate the usefulness of elastance in establishing PEEP in a protective, low V_T _strategy. The experimental protocol was designed to resemble a clinical procedure based on minimization of Ers, as used to set PEEP in patients with ALI [15,16,20]. PEEP titration with a protective low V_T _(ranging from 6 to 7 ml/kg) was performed in a swine oleic acid induced lung injury.

The main finding of our work is that optimization of PEEP based on minimizing the Ers appears to achieve the best compromise between recruitment/de-recruitment and hyperinflation. Additionally, as reported previously, tidal recruitment and hyperinflation appear to be simultaneous processes that occur in different lung regions during inspiration and at different PEEP levels [5,21,22].

After a recruitment manoeuvre, progressive reduction in PEEP from 26 cmH_2_O to PEEP_Ers _increased poorly aerated areas with a proportional reduction in hyperinflated areas; the distribution of normally aerated areas remained steady during this interval for all animals (Figures 2 to 4). It has been proposed that the amount of poorly aerated areas reflects the specific initial lesion; in oleic acid induced ALI, this is the capillary leakage with interstitial and alveolar oedema [23]. In view of this, high levels of PEEP appeared to reduce the amount of poorly aerated areas, probably by redistributing the interstitial oedema, but some of the normally aerated areas became hyperinflated.

PEEP_Ers _marked the pressure at which the coexistence of normally aerated, poorly aerated and hyperinflated areas appeared to minimize overall lung parenchyma recoil pressures, resulting in plateau pressures below 30 cmH_2_O (Table 1). The compromise achieved by PEEP_Ers_, resulting in a balance in the distribution of aeration, may be of value as a guide to mechanical ventilation and is in accordance with our recent findings obtained in healthy mechanically ventilated piglets, in which we used a similar protocol [17]. Comparing the dynamics of Ers and lung aeration at PEEP_Ers _with those at the highest PEEP step during the titration protocol, we identified a difference between healthy animal and those with induced ALI. In healthy piglets, a twofold rise in Ers was accompanied by a significant increment in hyperinflated areas and a concomitant reduction in normally aerated areas, suggesting direct correspondence between radiological evidence of hyperinflation and overstretching of the alveolar septum. In ALI conditions, a minor increase in hyperinflated areas and a steady amount of normally aerated areas were observed. Bearing this in mind, the increase in Ers in animals with ALI (from 54.5–81.5 cmH_2_O/l at PEEP_Ers _to 91–141.5 cmH_2_O/l at a PEEP of 26 cmH_2_O) may not solely be attributed to the increase in hyperinflated areas; it is possible that mechanical stress in alveolar septa at the interface of poorly aerated and non-aerated areas with normally aerated alveoli also played a role [4,9,24].

Another possibility is that an overall underestimation of aeration could occur as a consequence of the reduction in gas/tissue ratio in each voxel. The oleic acid induced injury produces acute endothelial and alveolar epithelial cell necrosis, resulting in multiple pulmonary microembolisms and protein-rich pulmonary oedema in a pattern that depends upon the distribution of perfusion [25-27]. Bearing these pathological mechanisms in mind, it is possible that an overall underestimation of aeration occurred, leading to an overestimation of non-aerated areas and therefore an underestimation of hyperinflated areas.

In the present study, a PEEP of 26 cmH_2_O appeared to prevent tidal de-recruitment (Figures 2 to 4). In agreement with our findings, Neumann and coworkers [28], using a similar model of ALI in pigs (weighing 31.3 ± 3.3 kg), found that oleic acid injured lungs tended to de-recruit rapidly during expiration when PEEPs lower than 15 cmH_2_O were applied, whereas PEEP levels greater than 20 cmH_2_O almost prevented tidal de-recruitment and PEEP at 25 cmH_2_O completely avoided cyclic de-recruitment/recruitment. It is therefore possible that a PEEP greater than PEEP_Ers _results in lung stability; however, this stability may be accompanied by overstretching caused by the hyperinflation of some previously normally aerated areas. Nevertheless, an analysis of the associated biological cost would be required to identify the potential benefits of this 'open the lung and keep it open' ventilatory strategy. Additionally, some lung units may only be recruited with hazardous levels of PEEP, which may have potential haemodynamic drawbacks, for instance the reduction in cardiac output related to a drop in preload caused by impaired venous return [24,29] and redistribution of blood flow away from well-ventilated units, which often increases ventilatory dead space [30].

In the present study it is reasonable to assume that PEEPs greater than 26 cmH_2_O would further increase the Ers, with a corresponding reduction in normally aerated and a steep increase in hyperinflated areas, in a pattern similar to that observed by Carvalho and coworkers [17] in healthy lungs at levels of PEEP in excess of PEEP_Ers_.

The institution of a PEEP level below PEEP_Ers _was associated with a progressive increase in non-aerated areas. A similar finding was described in a preceding report from our group [31], in which we proposed that PEEP_Ers _appears to prevent alveolar de-recruitment in ALI, according to analysis of CT scans. It is remarkable that the first step in PEEP below PEEP_Ers _resulted in an increase in poorly and non-aerated areas and a concomitant reduction in normally aerated areas in all animals studied (Figures 2 to 4). However, interpretation of these findings must take into account the inability of the CT morphological analysis to separate the effects of reduction in the amount of aeration from the concomitant increase in the amount of tissue and liquid observed with PEEP reduction.

The increase in the slice tissue mass as PEEP decreased, as well as from expiration to inspiration (Table 2), may reflect cephalo-caudal shrinking of the lungs or may result from the fact that, at high levels of PEEP, the V_T _may distribute outside the field of view of the CT scanner. However, we expect that a protective low V_T _would not cause enough displacement to move the area observed in the inspiratory slice beyond the block of expiratory slices. In fact, it was possible to recognize the same anatomical landmarks at end-expiration and end-inspiration images in all of the studied animals (Figures 2 to 5).

In accordance with our results, a reduction in lung mass as PEEP increased was reported by Karmrodt and coworkers [23]. Those authors compared the distribution of aeration in two experimental models of ALI (induced by oleic acid injection and surfactant depletion) in piglets (25 ± 1 kg). Different levels of continuous positive airways pressure were applied in a random order (ranging from 5 to 50 cmH_2_O), and CT scans of the whole lung were acquired at each level of continuous positive airways pressure (slice thickness 1 mm). The volume of lung tissue decreased from 223 ± 53 ml to 35 ± 17 ml at a continuous positive airways pressure of 5 and 50 cmH_2_O, respectively, mainly in poorly aerated and non-aerated compartments.

In pigs with ALI induced by surfactant depletion, Suarez-Sipmann and coworkers [18] recently reported that continuous monitoring of dynamic compliance allowed detection of the beginning of lung collapse during descending titration of PEEP. The authors reported that the PEEP at which maximal compliance was observed was between 16 and 12 cmH_2_O in all eight studied animals, and that a PEEP of 16 cmH_2_O was required to prevent lung de-recruitment, achieving a compromise between mechanical stress, intrapulmonary shunt and PaO_2_. Thus, low PEEP levels increased Ers by several mechanisms, such as reduction in lung aerated volume as a consequence of alveoli flooding by haemorrhagic oedema in dependent regions, and tidal overstretching of some previously normally aerated areas, especially in nondependent regions. These mechanical effects may be accompanied by a progressive reduction in PaO_2 _and augmented intrapulmonary shunt, as shown by Suarez-Sipmann and coworkers [18].

The airways resistance exhibited dynamics similar to those of Ers during PEEP titration. With progressive reduction in PEEP from 26 cmH_2_O to ZEEP, the airways resistance exhibited a smooth reduction until PEEP_Ers _was reached, after which it rose again, showing marked augmentation between PEEP at 6 cmH_2_O and ZEEP. At low levels of PEEP, the augmentation in Rrs may be attributed to progressive closure of the airways; however, clearance of mucus during the reduction in PEEP could have contributed to the elevation in Rrs. The higher values of Rrs at PEEP levels greater than PEEP_Ers _were unexpected, and one may speculate that it may have been caused by uneven distribution of ventilation as a consequence of reduced regional compliance in hyperinflated areas. Additionally, the hyperinflated areas at nondependent lung regions may compress dependent lung regions, contributing to a heterogeneous distribution of ventilation, as proposed by Suarez-Sipmann and coworkers [18].

The use of descending PEEP titration after a recruitment manoeuvre to minimize Ers may be a practical approach to establishing PEEP during controlled mechanical ventilation. Ward and colleagues [15] showed that the process of selecting PEEP based on minimizing the Ers may be easier and could be more frequently applied at the bedside than use of a static pressure-volume curve. However, as described by Suter and coworkers [32], the pressure at minimal Ers is dependent and decreases with increasing V_T_. This volume dependence of Ers could be minimized by using a fixed small V_T _(such as 5 to 6 ml/kg) during the titration protocol. This V_T _range is in accordance with the current recommendations for a protective ventilatory strategy [2,33] and is essential to minimize dependence of Ers on V_T _and to prevent adjustment of PEEP to an inadequate level.

The benefits of instituting high levels of PEEP appear to depend on the pattern of lung injury distribution [4]. Our findings recapitulate the radiological appearance of a diffuse pattern of ALI/acute respiratory distress syndrome, which has high recruitment potential [21,34]. Further research may be required to determine the correspondence between Ers dynamics and the pattern of aeration in lungs with a focal distribution, which have low recruitment potential and a large amount of normally aerated areas [21,34].

In summary, continuous monitoring of the Ers, estimated using least-squares linear regression, during a descending PEEP titration after a recruitment manoeuvre apparently indicates that PEEP_Ers _represents a balance between lung aeration and mechanical stress. These findings support the proposal that this technique, which is feasible at the bedside, may help to prevent lung de-recruitment [18] and minimizes the coexistence of poorly aerated and hyperinflated areas.

### Study limitations

A limitation of the present study is that the lung morphological analysis was based on a single slice of the CT scan taken at the juxta-diaphragmatic level. One could question whether such an image is truly representative of the whole lung. However, it could be argued that the amount of non-aerated areas is likely to be well represented, because these areas are more common near to the diaphragm [33,35]. The distribution of hyperinflated areas appears to represent a discrete cephalo-caudal gradient, as shown in Figure 5. It can also be observed that the apex distribution of aeration was similar to the distribution at the juxta-diaphragmatic level. The middle level (close to the carina) exhibited fewer hyperinflated areas than the apex and basal levels in one animal, which is probably attributable to the presence of the heart-limiting lung expansion in nondependent regions [36]. Our data are in accordance with findings recently reported by Karmrodt and coworkers [31]. In pigs with ALI induced by oleic acid, those investigators described only a small cephalo-caudal gradient of hyperinflated areas at different levels of continuous positive airways pressure.

The blood gas analyses were not conducted during PEEP titration at each PEEP step. However, several studies suggest that the amount of alveolar flooding, observed during morphological analyses of the CT scan images, exhibits an inverse correlation with PaO_2 _dynamics [28,37,38]. Additionally, in surfactant-depleted piglets, Suarez-Sipmann and coworkers [18] showed that as PEEP is reduced after a recruitment manoeuvre, the PaO_2_/fractional inspired oxygen ratio decreased with a concomitant augmentation of intrapulmonary shunt fraction.

The effects of chest wall elastance were not measured in the present study. However, in a similar model of ALI in piglets, de Abreu and coworkers [39] showed that the chest wall elastance made just a small contribution to the overall properties of the respiratory system. Additionally, the effects of the nonphysiological supine position on the overall distribution of aeration in piglets were not assessed in the present study. However, it is expected that, as the lung injury was induced with the animals in supine position, the primary lesion was more likely to have occurred in dependent regions. In accordance with this, Karmrodt and coworkers [31] showed, in a similar oleic acid injury model, that non-aerated areas were predominantly located in these dependent regions, and that the weight of the heart also contributes to lung collapse in caudal regions. Furthermore, those authors described a decrease in non-aerated lung volume along the cranio-caudal axis at high levels of airway pressure. These findings are apparently in accordance with our data as well as with the effects of PEEP on regional distribution of aeration in humans [21].

The temporal effect on lung stability was not accessed in the present study. However, alveolar de-recruitment in oleic acid injury models seems to occur during the first few moments of expiration [28,29]. Based on this, we believe that complete stabilization of lung compartments should have occurred by the end of each PEEP step. Additionally, PEEP_Ers _obtained in our protocol was near to that obtained in the work reported by Suarez-Sipmann and coworkers [18], which used a 10 min time interval for each PEEP step. In the present protocol, we attempted to achieve a compromise was between the PEEP step time interval and the total time required to perform the entire PEEP titration, in order to make this manoeuvre useful in clinical practice.

## Conclusion

In an porcine model of ALI induced by oleic acid PEEP_Ers_, obtained after a recruitment manoeuvre followed by descending PEEP titration, corresponded to the highest amount of normally aerated areas, with less poorly aerated and hyperinflated areas, according to CT scan morphologic analysis. The institution of high levels of PEEP reduced the poorly aerated areas but also enlarged the hyperinflated areas. The reduction in PEEP consistently increased poorly or non-aerated areas as well as tidal re-aeration, especially at low PEEP (PEEP < 6 cmH_2_O). Hence, the PEEP_Ers _may be a useful aid to optimizing lung aeration to minimize lung mechanical stress.

## Key messages

• Administration of PEEP, downward titrated after a recruitment manoeuvre, may prevent cyclic recruitment/de-recruitment.

• PEEP_Ers _represented a compromise between maximizing normally aerated areas and minimizing tidal recruitment and hyperinflation.

• Lung stability may be obtained with high levels of PEEP at the expense of hyperinflation of previously normally aerated areas.

• The Ers is a feasible bedside index and may be useful in selecting a PEEP level that balances lung aeration and mechanical stress in lung injury.

## Abbreviations

ALI = acute lung injury; CT = computed tomography; Ers = elastance of the respiratory system; PEEP = positive end-expiratory pressure; PEEP_Ers _= PEEP at which the minimum Ers was observed; Rrs = resistance of the respiratory system; V_T _= tidal volume; ZEEP = zero end-expiratory pressure.

## Competing interests

The authors declare that they have no competing interests.

## Authors' contributions

ARSC, FCJ, FAB, FOA and JS participated in the design of the study and carried out the experiments. ARSC processed the data, performed the statistical analysis and wrote the manuscript. AVP designed the experimental setup. RR established the CT protocol and analysis. AGN and FCJ conceived and coordinated the study, and helped to write the manuscript. All authors read and approved the final manuscript.
